# Pentagamavunon-1 (PGV-1) inhibits ROS metabolic enzymes and suppresses tumor cell growth by inducing M phase (prometaphase) arrest and cell senescence

**DOI:** 10.1038/s41598-019-51244-3

**Published:** 2019-10-16

**Authors:** Beni Lestari, Ikuko Nakamae, Noriko Yoneda-Kato, Tsumoru Morimoto, Shigehiko Kanaya, Takashi Yokoyama, Masafumi Shionyu, Tsuyoshi Shirai, Edy Meiyanto, Jun-ya Kato

**Affiliations:** 1 Laboratory of Tumor Cell Biology, Division of Biological Science, Graduate School of Science and Technology, Nara Institute of Science and Technology, Nara, Japan; 2 Laboratory of Synthetic Organic Chemistry, Division of Materials Science, Graduate School of Science and Technology, Nara Institute of Science and Technology, Nara, Japan; 3 Laboratory of Computational Systems Biology, Division of Information Science, Graduate School of Science and Technology, Nara Institute of Science and Technology, Nara, Japan; 4 Nagahama Institute of Bio-Science and Technology, Nagahama, Japan; 5 Cancer Chemoprevention Research Center, Faculty of Pharmacy, Universitas Gadjah Mada, Yogyakarta, Indonesia

**Keywords:** Cancer, Molecular medicine, Drug development

## Abstract

We previously showed that curcumin, a phytopolyphenol found in turmeric (*Curcuma longa*), targets a series of enzymes in the ROS metabolic pathway, induces irreversible growth arrest, and causes apoptosis. In this study, we tested Pentagamavunon-1 (PGV-1), a molecule related to curcumin, for its inhibitory activity on tumor cells *in vitro* and *in vivo*. PGV-1 exhibited 60 times lower GI_50_ compared to that of curcumin in K562 cells, and inhibited the proliferation of cell lines derived from leukemia, breast adenocarcinoma, cervical cancer, uterine cancer, and pancreatic cancer. The inhibition of growth by PGV-1 remained after its removal from the medium, which suggests that PGV-1 irreversibly prevents proliferation. PGV-1 specifically induced prometaphase arrest in the M phase of the cell cycle, and efficiently induced cell senescence and cell death by increasing intracellular ROS levels through inhibition of ROS-metabolic enzymes. In a xenograft mouse model, PGV-1 had marked anti-tumor activity with little side effects by oral administration, whereas curcumin rarely inhibited tumor formation by this administration. Therefore, PGV-1 is a potential therapeutic to induce tumor cell apoptosis with few side effects and low risk of relapse.

## Introduction

Tumor cells arise from normal cells by a series of genetic mutations, which confer various tumor-specific features, including unregulated cell proliferation, impaired cell differentiation, resistance to cell death and senescence, and reduced genomic stability^1,2^. Therefore, these mutated gene products are a good target for cancer therapy, and a number of targeted drugs against these gene products have been developed. The first type of this drug was imatinib mesylate (Gleevec, also known as STI–571) for chronic myelogenous leukemia (CML)^3,4^, which was followed by various drugs including tyrosine kinase inhibitors, Raf inhibitors, MEK inhibitors, CDK4/6 inhibitors, and monoclonal antibodies specifically recognizing receptors on the cancer cell surface.

The effect of STI-571 on CML is superb. The eight-year survival of patients treated with STI-571 is 85%, the complete hematologic response (CHR) is 98%, the complete cytogenetic response (CCyR) is 87%, and 93% of the patients can be maintained in the chronic phase without proceeding to the transition phase or acute phase. However, Gleevec cessation causes relapse in 61% of CML patients^5^, which suggests that, even though it is very effective, STI-571 is not an ultimate cure, and more novel drugs are still needed.

Curcumin is one of the main phytopolyphenol component in turmeric (*Curcuma longa*), and exhibits anti-cancer activity against many types of human cancer cells^6,7^. Forty-four clinical trials and 5 animal experiments showed therapeutic benefits^8^. Furthermore, treatment with curcumin alone prevented relapse of multiple myeloma in a patient who repeatedly suffered relapse of this disease^9^. Therefore, the anti-tumorigenic molecular action of curcumin warrants further investigation. Although many pathways and factors link curcumin to anti-tumorigenic activity^7^, how curcumin precisely suppresses tumors remained to be clarified.

We previously found^10^ that curcumin specifically interacts with several ROS-metabolic enzymes^11^, such as NAD(P)H dehydrogenase [quinone] 1 (NQO1), carbonyl reductase 1 (CBR1), glyoxalase I (GLO1), glutathione-S-transferase phi 1 (GST-P1), and aldo-keto reductase family 1 member 1 (AKR1C1). Treatment with curcumin increases intracellular ROS levels in leukemic cells, and antioxidant reversed the effect of curcumin. Furthermore, curcumin irreversibly arrests the cell cycle and induces cellular senescence in cultured leukemic cells in an antioxidant-sensitive manner, and effectively suppresses tumor formation in a xenograft mouse model^10^. The control of intracellular ROS levels is a promising approach for tumor suppression^12,13^, and curcumin is a potential candidate for this purpose. However, some properties of curcumin are not necessarily optimal for a drug in humans; curcumin has low solubility in water, low absorbance from the gut, and low stability *in vivo*^14,15^. Therefore, derivative drugs and curcumin analogs, which improve these deficiencies, are needed.

Here, we analyzed the anti-tumorigenic activity of a monocarbonyl curcumin analogue, pentagamavunon-1 (PGV-1)^16^. PGV-1 was superior to curcumin in several ways; (1) irreversible growth suppression of human tumor cell lines at a lower dose in an *in vitro* culture system, (2) efficient induction of cellular senescence and death, and (3) tumor suppression in a xenograft mouse model via p.o. In addition, PGV-1 specifically induced prometaphase arrest in M phase, whereas curcumin has an inhibitory effect at multiple points in the cell cycle. PGV-1 interacted with a subset of curcumin-interacting ROS-metabolic enzymes, including GST-P1, NQO1, NQO2, GLO1, and AKR1C1, and inhibited their enzymatic activities by competing with their co-factors. These results indicate that PGV-1 is a good anti-cancer drug candidate.

## Results

### Anti-proliferative activity of PGV-1 in leukemic cells *in vitro*

**Figure 1:**
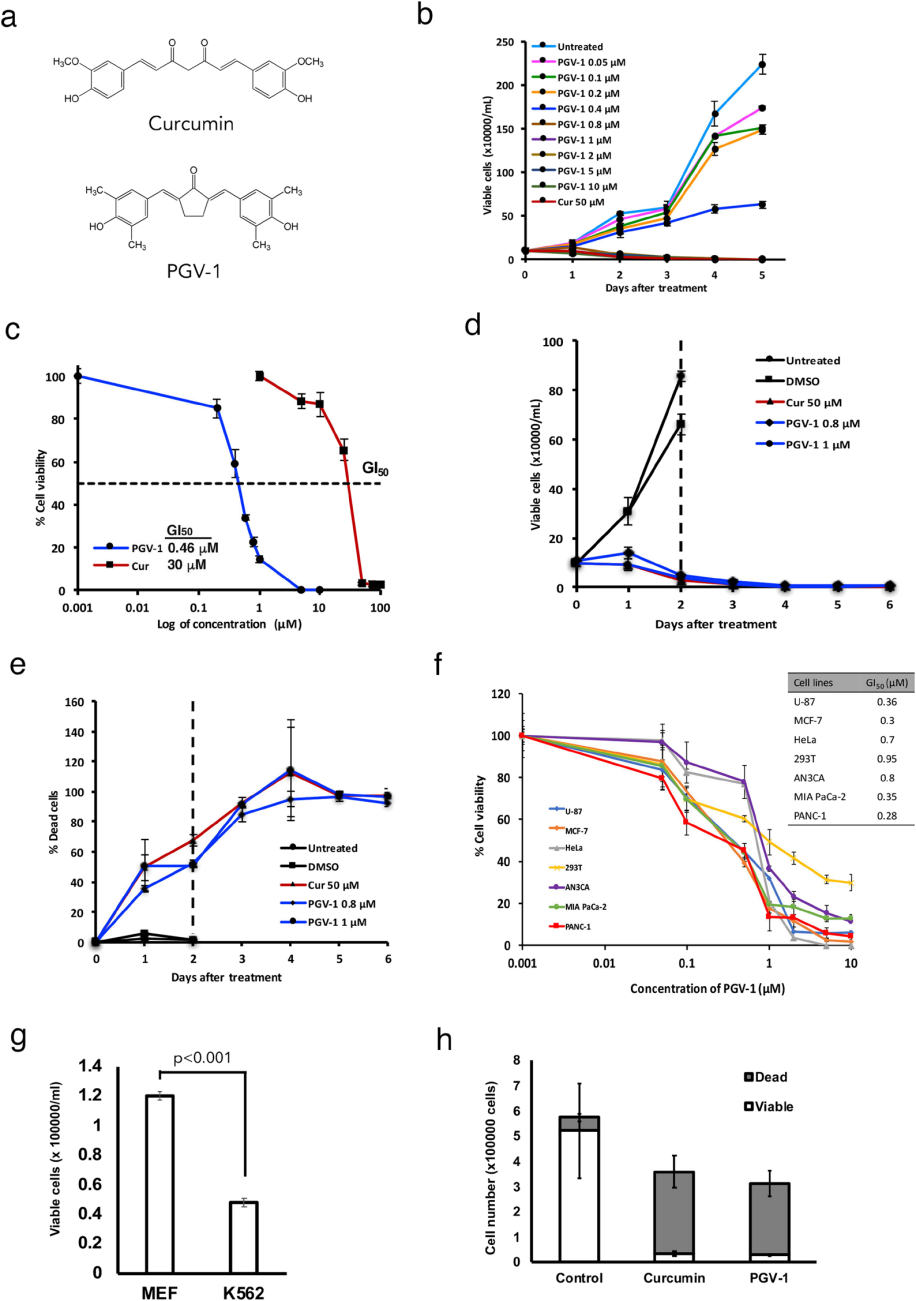
PGV-1 suppresses tumor cell growth *in vitro*. (**a**) Chemical structure of curcumin and PGV-1. (**b**) K562 cells (1 × 10^5^ cells/mL) were maintained with curcumin (50 μM) and PGV-1 (0.05–10 μM) for 5 days and enumerated every day after trypan blue staining. (**c**) The GI_50_ of curcumin and PGV-1 on K562 cells was determined after a 4-day culture. (**d**,**e**) K562 cells were treated with curcumin (50 μM) and PGV-1 (0.8 and 1 μM) for 2 days, washed once and transferred to the fresh medium without either compound. Viable and dead cells were counted by the trypan blue exclusion method at the indicated times. The number of viable cells (**d**) and dead cells (**e**) are shown. (**f**) U-87 MG, MCF-7, HeLa, 293T, AN3CA, Mia Paca-2, and PANC-1 cells were treated with PGV-1 for 4 days, viable cells were counted by the trypan blue exclusion assay, and the GI_50_ of PGV-1 was determined. The results in panels B-F were the average of three independent experiments (means ± SD). (**g**) MEF and K562 cells (1 × 10^5^ cells/mL) were cultured in the presence of PGV-1 (0.8 and 1 μM) and counted after 2 days using the trypan blue staining method. (**h**) K562 cells (2.5 × 10^6^ cells) were subcutaneously injected into the flanks of mice, which had been treated with curcumin and PGV-1 (20 mg/kg BW) in corn oil via intraperitoneal administration. After 2 days, mice were sacrificed, tumor cells were taken, and counted using the trypan blue staining method.

To examine the anti-proliferative activity of PGV-1, a monocarbonyl analogue of curcumin^16^, (Fig. 1a), we incubated leukemic K562 cells in the absence and presence (from 0.05 to 10 μM) of PGV-1 *in vitro* (Fig. 1b). Compared to curcumin (50 μM) used as a control, PGV-1 higher than 0.4 μM significantly suppressed the growth of K562 cells. We next determined and compared the GI_50_ of PGV-1 and curcumin using K562 cells (Fig. 1c). The GI_50_ of PGV-1 and curcumin were 0.46 and 30 μM, respectively, indicating that PGV-1 was more than 60 times more inhibitory than curcumin in this assay.

Because we previously reported that curcumin irreversibly inhibits leukemic cell growth^10^, we examined the effect of PGV-1 after its removal from the medium. For this purpose, we cultured K562 cells in the presence of PGV-1 and curcumin for 2 days. After washing, cells were maintained in fresh medium without either drug for up to six days, and both dead and alive cells were counted (Fig. 1d,e). Because PGV-1 is ca 60-fold superior to curcumin, 0.8 and 1 μM of PGV-1 were used. Figure 1d,e show that cells treated with either PGV-1 or curcumin remained growth-inhibited and gradually lost viability even in the absence of the drug (viability was determined by the trypan blue exclusion method).

The growth inhibitory effect of PGV-1 was not restricted to K562 leukemic cells. Figure 1f shows that other types of human cancer cell lines including U-87 MG glioblastoma, MCF-7 breast adenocarcinoma, HeLa cervical cancer, AN3CA uterine cancer, MIA PaCa-2 and PANC-1 pancreatic cancer, and 293T human embryonic kidney cells, were sensitive to the inhibition of growth by PGV-1. The GI_50_ value varied between 0.28 and 0.95 depending on the cell line used, and we concluded that PGV-1 effectively inhibited the proliferation of human cancer cells with low GI_50_.

To evaluate the cytotoxic effect of PGV-1 on normal cells, we used very early-passage (p3) mouse embryonic fibroblasts (MEFs), which are devoid of any genomic damages. MEFs and K562 cells were cultured in the presence of PGV-1 (0.8 μM), and viable cells were counted after 2 days. Figure 1g shows that a significant number of MEF cells remained viable after PGV-1-treatment, whereas K562 cells markedly reduced their viability by PGV-1 as expected from the results shown in Fig. 1b,d.

We next examined the anti-proliferative effect of curcumin and PGV-1 *in vivo*. K562 cells were subcutaneously injected into the flanks of nude mice, which had been intraperitoneally administrated with curcumin and PGV-1 dissolved in corn oil (20 mg/kg BW). After 2 days, cells were recovered from mice, and both dead and viable cells were counted after trypan-blue staining (Fig. 1h). Under these conditions, both curcumin and PGV-1 effectively suppressed proliferation of tumor cells *in vivo*.

### PGV-1 induces prometaphase arrest in M phase of the cell cycle and subsequent cell senescence and cell death

**Figure 2:**
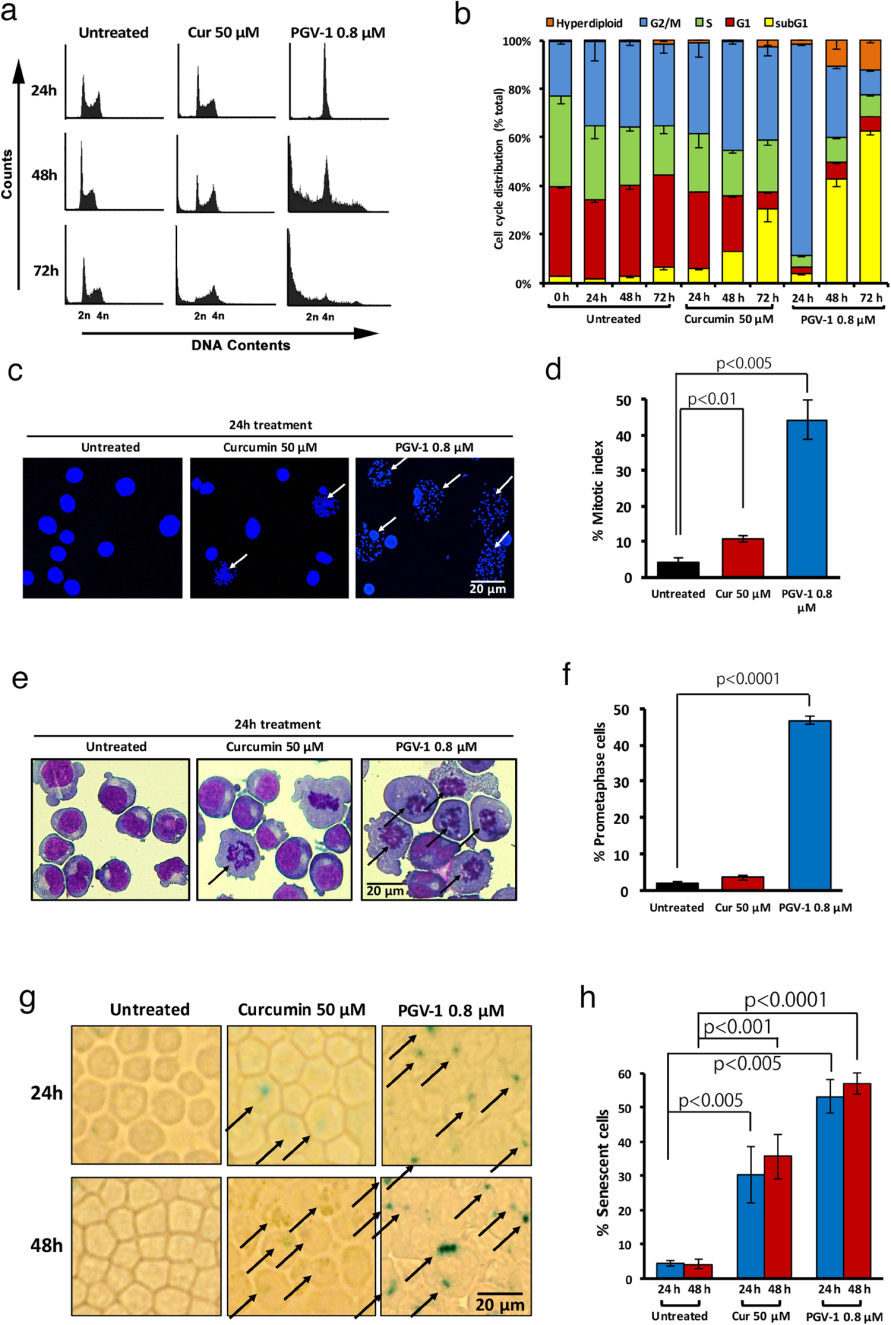
Effects of curcumin and PGV-1 on tumor cell proliferation *in vitro*. (**a**) K562 cells (5 × 10^5^ cells/mL) were treated with curcumin (50 μM) and PGV-1 (0.8 μM) for 24, 48, and 72 hr, and then subjected to cell cycle analysis. (**b**) The percentage of cells in each phase as in panel A is shown as the mean ± SD from three independent experiments. (**c**) Mitotic spread of K562 cells treated with curcumin (50 μM) and PGV-1 (0.8 μM) for 24 hr. Chromosomal DNA was stained with Hoechst 33342 and viewed by confocal microscopy. Cells with no nuclear envelope are marked by arrows. The scale bar indicates 12.5 μm. (**d**) Mitotic indices calculated from three independent experiments as in panel c are shown as means ± SD. (**e**) K562 cells treated with curcumin (50 μM) and PGV-1 (0.8 μM) for 24 hr were stained with a May-Grunwald Giemsa solution. Cells with no nuclear envelope and condensed chromosomes that were not aligned along the metaphase plate are marked by arrows. The scale bar indicates 12.5 μm. (**f**) The percentage of prometaphase cells was calculated from three independent experiments as in panel e and is shown as the mean ± SD. (**g**) K562 cells treated with curcumin (50 μM) and PGV-1 (0.8 μM) for 24 and 48 hr were harvested on the cover glass by cytospin and stained for the β-galactosidase activity. Cells with positive SA-β-gal signals are marked by arrows. The scale bar indicates 50 μm. (**h**) The percentage of senescent cells (β-galactosidase-positive cells) was calculated. (n = 3).

To better understand the anti-proliferative activity of PGV-1, K562 cells were incubated with PGV-1 (0.8 μM) and curcumin (50 μM) for 1–3 days and subjected to cell cycle analysis. Figure 2a,b (see Supplementary Fig. S1 for the cell cycle profile in earlier time points) show that exposure to curcumin gradually accumulated cells in G2/M phase of the cell cycle (from 22.6 ± 1.0% at 0 hr to 37.6 ± 5.6% at 24 hr and 44.4 ± 1.0% at 48 hr) and underwent cell death (from 2.97 ± 0.27% at 0 hr to 30.2 ± 5.3% at 72 hr, represented by the subG1 population), whereas cells treated with PGV-1 rapidly increased the population in G2/M phase within 24 hr (from 22.6 ± 1.0% at 0 hr to 87.3 ± 0.47% at 24 hr) and subsequently induced rapid cell death (from 2.97 ± 0.27% at 0 hr to 40.1 ± 2.9% at 48 hr and 64.3 ± 1.5% at 72 hr), indicating that PGV-1 has increased anti-tumor cell growth activity compared to that of curcumin. Treatment with PGV-1 increased the population of cells with ploidy higher than 4n (from 0.48 ± 0.3% at 0 hr to 12.3 ± 1.0% at 72 hr), which suggests that some cells bypassed the G2/M block and reinitiated DNA replication.

To further determine the precise arrest point in the cell cycle by PGV-1, we measured the mitotic index. K562 cells were treated with PGV-1 (0.8 μM) and curcumin (50 μM) for 24 hr, and subjected to the mitotic spread assay in which M phase cells can be discriminated from G1/S/G2 cells by the disappearance of the nuclear membrane. Figure 2c,d show that treatment with PGV-1 markedly increased the number of cells with nuclear envelope breakdown (4.19 ± 1.1% for untreated vs 44.3 ± 5.5% for PGV-1 treatment), indicating that PGV-1 arrested cells largely in M phase. Next, we immobilized cells treated with PGV-1 and curcumin for 24 hr on a glass plate by cytospin and stained cells in a Giemza solution. Figure 2e,f show that cells with no nuclear envelope by PGV-1 treatment had condensed chromosomes that were not aligned along the metaphase plate nor separated toward the two poles, which suggests that cells were arrested in the prometaphase stage of M phase (1.94 ± 0.37% for untreated vs 46.7 ± 1.0% for PGV-1 treatment).

Because we previously showed that curcumin induced cell senescence and cell death^10^, we investigated whether PGV-1 has similar activity. For this purpose, cells were treated with PGV-1 and curcumin for 24 hr and 48 hr, and tested for senescence associated (SA)-β-galactosidase (gal) activity, a marker of senescence. Figure 2g shows that treatment with PGV-1 and curcumin induced SA-β-gal activity, but quantification of SA-β-gal-positive cells (Fig. 2h) indicated that PGV-1 induced an SA-β-gal expression more rapidly (30.4 ± 8.2% for curcumin at 24 hr versus 53.3 ± 5.0% for PGV-1 at 24 hr) and more efficiently (35.8 ± 6.4% for curcumin at 48 hr versus 56.9 ± 3.1% for PGV-1 at 48 hr) than curcumin. Furthermore, as shown in Fig. 1d, cells treated with PGV-1 for 2 days did not reenter the cell cycle even after removing PGV-1 from the medium. These two lines of evidence indicate that PGV-1 induced senescence to a greater extent than curcumin. Thus, the action of PGV-1 may be as follows: in the first 24 hr, PGV-1 arrests cells at prometaphase and induces senescence, and in the next 24–48 hr, PGV-1 induces cell death with kinetics faster than that of curcumin.

### PGV-1 binds to a subset of curcumin-interacting ROS-metabolic enzymes

**Figure 3:**
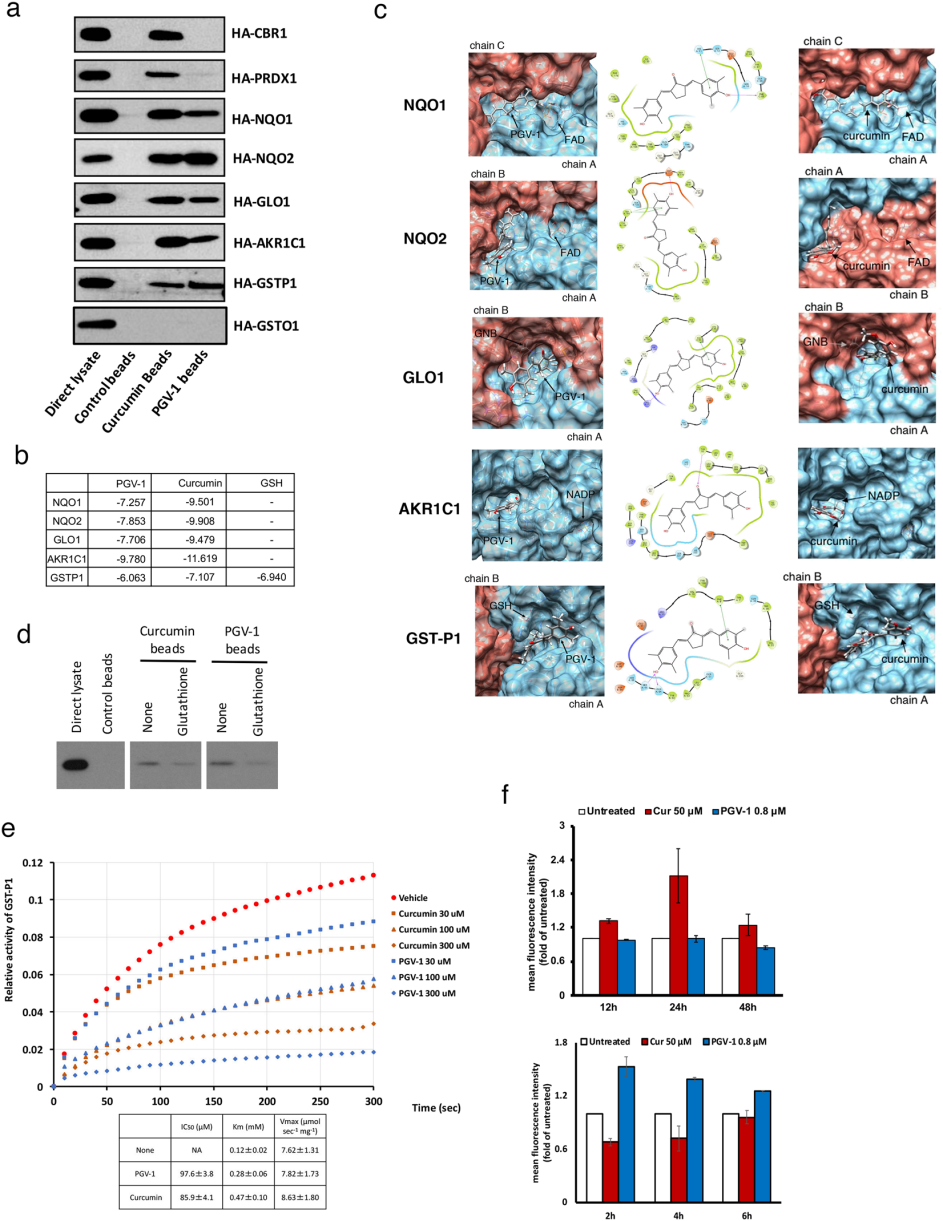
PGV-1 inhibits ROS metabolic enzymes. (**a**) Control, curcumin, and PGV-1 beads were subjected to a pull-down assay using cell lysates containing HA-CBR1, HA-PRDX1, HA-NQO1, HA-NQO2, HA-GLO1, HA-AKR1C1, HA-GSTP1, and HA-GSTO1 proteins. Bound proteins were visualized by immunoblotting using an anti-HA antibody. (**b**) Docking scores between ROS scavengers and PGV-1 or curcumin. (**c**) Docking poses between ROS scavengers and PGV-1 (left panels) or curcumin (right panels). Positions of the bound co-factors (FAD, GNB, NADP, and GSH) are also shown. Schematic diagrams of binding between PGV-1 and the corresponding amino acids are shown in middle panels. (**d**) Competitive pulldown assay between 293T cell lysates containing HA-GSTP1 and curcumin/PGV-1 beads in the presence or absence of 10 mM glutathione. Bound proteins were visualized by immunoblotting using an anti-HA antibody. (**e**) GST-P1 enzymatic activity was measured *in vitro* in the presence of curcumin and PGV-1. The IC_50_ of each compound is shown as the mean ± SD. Km and Vmax were also calculated. (**f**) K562 cells treated with curcumin (50 μM) and PGV-1 (0.8 μM) for 12, 24 and 48 hr (upper panel), or for 2, 4, and 6 hr (lower panel), were subjected to the ROS detection analysis using FACS.

We previously showed^10^ that curcumin binds to a group of ROS metabolic enzymes^11^. Therefore, we investigated whether PGV-1 interacts with these enzymes *in vitro*. We immobilized PGV-1 and curcumin on epoxy-sepharose beads, and incubated with lysates isolated from cells transfected with HA-tagged enzymes. Figure 3a shows that PGV-1 interacted with NAD(P)H dehydrogenase [quinone] 1 and 2 (NQO1 and NQO2), glyoxalase I (GLO1), aldo-keto reductase family 1 member 1 (AKR1C1), and glutathione-S-transferase phi 1 (GST-P1) but little with carbonyl reductase 1 (CBR1) or peroxiredoxin-1 (PRDX1) under these conditions. In addition, because human cells contain several GST family proteins^17^, we cloned another GST gene, GST omega 1 (GST-O1), and tested whether it bound to curcumin and PGV-1. We found that GST-O1 did not bind to either, which suggests that there is a high specificity of interaction between curcumin/PGV-1 and the GST family of proteins.

To obtain insights into the molecular action of PGV-1 on ROS metabolic enzymes, we performed a molecular docking analysis. Figure 3b shows the docking scores between ROS metabolic enzymes and curcumin/PGV-1, and Fig. 3c shows the docking poses between the enzymes and PGV-1/curcumin, which suggests that the most probable binding site is located near the region required for co-factor binding. This result suggests that PGV-1 and curcumin compete with co-factors, such as FAD, GNB, NADP, or GSH, for binding to ROS metabolic enzymes. For example, the docking scores between GST-P1 and curcumin/PGV-1 were −7.107/−6.063, respectively, whereas the score between GST-P1 and GSH was −6.940, which implies that curcumin/PGV-1 binds to GST-P1 with comparable affinity to that of co-factors. Furthermore, molecular docking analysis (Fig. 3c) suggests that Tyr7 and Asp98, which are required for the enzymatic activity and interaction with GSH, respectively (UniProt database), are involved in the interaction with PGV-1.

To further understand how curcumin/PGV-1 competes with GSH for binding to GST-P1, we performed pulldown assays using PGV-1/curcumin-beads and lysates containing HA-tagged GST-P1 in the presence or absence of glutathione, a co-factor for GST proteins^17^. Figure 3d shows that the interaction between PGV-1/curcumin and GST-P1 was inhibited by a high concentration of glutathione (10 mM). In addition, we examined the effect of PGV-1 and curcumin on the enzymatic activity of GST-P1^18^ (Fig. 3e). For this assay, GST-P1 proteins were expressed in *E. coli* and affinity-purified. Purified recombinant protein was incubated with a reduced form of glutathione (GSH) and 1-chloro-2,4-dini-trobenzene (CDNB), and the amount of GSH-conjugated CDNB was detected by monitoring the absorbance at 340 nm. Figure 3e shows that both curcumin and PGV-1 inhibited the activity of GST-P1 with an IC_50_ of 85.9 ± 4.1 μM and 97.6 ± 3.8 μM, respectively. Using this assay, we also calculated the Km and Vmax of GST-P1 as 0.12 ± 0.02 mM and 7.62 ± 1.31 μmol sec^−1^ mg^−1^, respectively. We further found that the Km and Vmax in the presence of curcumin and PGV-1 were 0.47 ± 0.10 mM and 8.63 ± 1.80 μmol sec^−1^ mg^−1^ for curcumin, and 0.28 ± 0.06 mM and 7.82 ± 1.73 μmol sec^−1^ mg^−1^ for PGV-1, respectively. Because PGV-1 had limited effect on the Vmax but increased the Km more than 2 fold, PGV-1 seems to act as a competitive inhibitor. Thus, PGV-1 inhibited the enzymatic activities of ROS scavengers by competing with co-factors at the binding site.

Finally, we investigated whether PGV-1 increases intracellular ROS levels. Curcumin increases ROS levels 24 hr after addition of curcumin into the medium^10^, but we did not detect an increase of ROS levels in cells treated with PGV-1 after 12, 24 and 48 hr (Fig. 3f, upper panel). Therefore, we measured ROS levels at a much earlier time point (Fig. 3f, lower panel), and found that PGV-1 increased ROS levels after 2 hr, but curcumin did not. Thus, we concluded that PGV-1 binds to ROS metabolic enzymes, including NQO1, NQO2, GLO1, AKR1C1, and GST-P1, inhibits their enzymatic activities by competing with co-factors, and increases intracellular ROS levels earlier than that of curcumin.

### Anti-tumorigenic activity of PGV-1 in a mouse xenograft model

**Figure 4:**
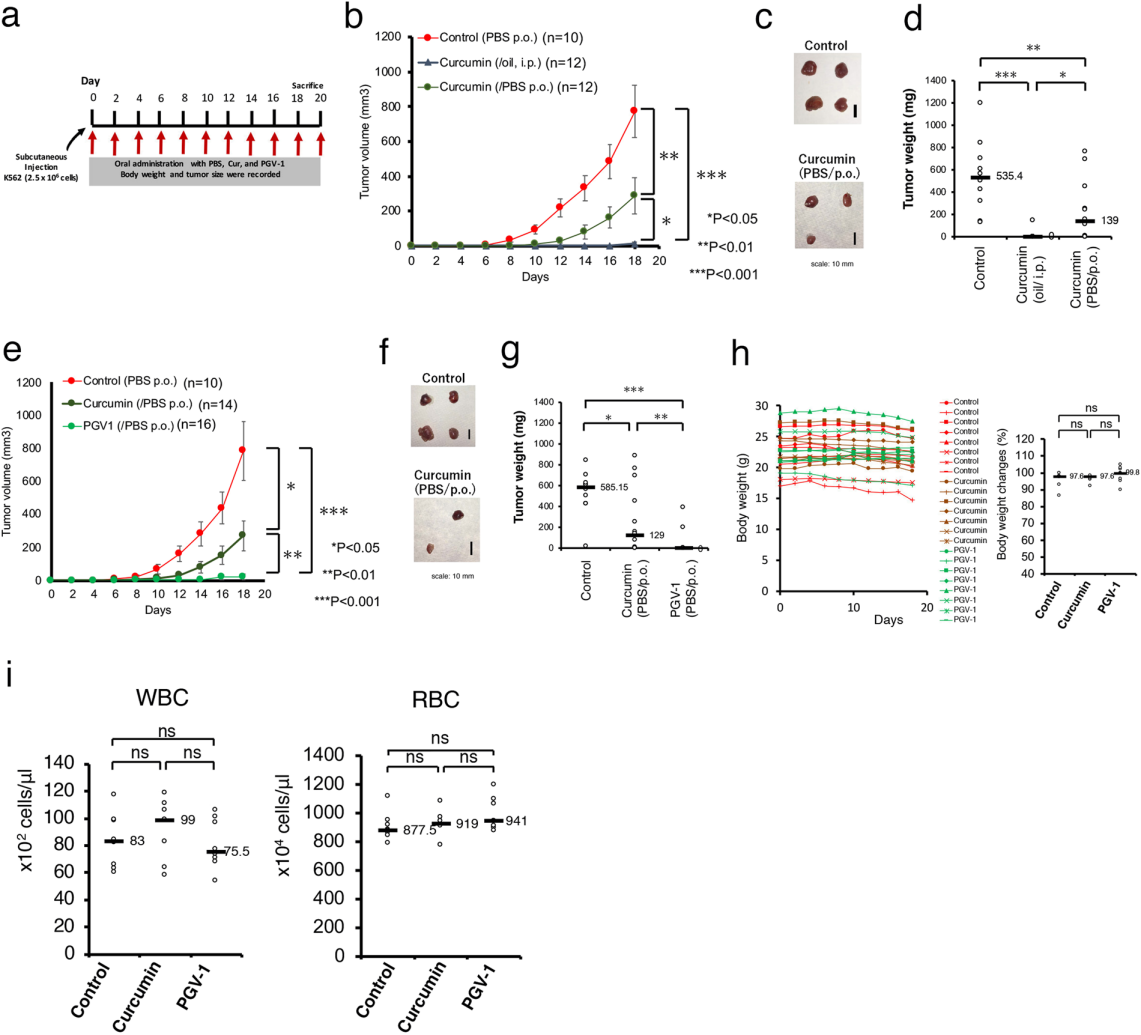
PGV-1 suppresses tumor formation *in vivo*. (**a**) Experimental design of testing for anti-tumor activity of curcumin and PGV-1. (**b**-**d**) K562 cells (2.5 × 10^6^ cells) were transplanted s.c. into the flanks of mice. Mice were then treated every 2 days with curcumin (20 mg/kg BW) in corn oil via i.p., curcumin in PBS via p.o., and control (PBS, p.o.). Tumor sizes were measured every 2 days during this period (**b**). After 18 days, mice were sacrificed, tumors were taken (**c**), and tumor weights were measured (**d**). (**e**-**i**) K562 cells (2.5 × 10^6^ cells) were transplanted s.c. into the flanks of mice. Mice were then treated every 2 days with curcumin and PGV-1 in PBS via p.o., and control (PBS, p.o.). The tumor sizes were measured every 2 days (**e**). After 18 days, mice were sacrificed and tumors were taken (**f**) and measured (**g**). The body weights were measured every 2 days (**h**, left panel), and, after 18 days, they were compared (**h**, right panel). The white and red cells in peripheral blood were counted at the time of sacrifice (**i**). Data are shown as means ± SD. In panels D, G, H, and I, medians are also shown. ns; not significant.

Curcumin suppressed the tumorigenic cell growth of human cancer cells in a xenograft mouse model (Fig. 4a). We confirmed its anti-tumorigenic activity by intraperitoneal injection^10^ and tested different curcumin administration method in K562 leukemic cells injected into nude mice (Fig. 4b–d). We found that intraperitoneal (i.p.) injection of curcumin dissolved in corn oil markedly suppressed tumor formation in this xenograft mouse model, whereas oral administration (per os, p.o.) of curcumin dissolved in PBS was less effective.

Next, nude mice were subcutaneously (s.c.) injected with K562 cells, and PGV-1 dissolved in PBS was administered to these mice via p.o. every 2 days for 20 days (Fig. 4a). As controls, PBS alone and curcumin in PBS were given. After 18–20 days, tumors were visible at all injection sites in control mice, and treatment with curcumin via p.o. partially suppressed formation of tumors, whereas little tumors were detected in mice administrated with PGV-1 via p.o. (Fig. 4e–g).

PGV-1-treated mice exhibited little decrease in body weight (Fig. 4h), nor a decrease in white and red blood cell counts in peripheral blood (Fig. 4i), nor any other effects in behavior and macroscopic appearance. Thus, PGV-1 was sufficiently potent to suppress tumor formation *in vivo*, but exhibited little or no obvious adverse effects on the normal lineage of cells.

## Discussion

Curcumin has tumor suppressing activity in various human tumor cells *in vitro* and *in vivo*^6,7^. However, because of limitations in the properties of curcumin, which include low solubility in water, low absorbance from the gut, and low stability *in vivo* etc.^14,15^, curcumin has never been a versatile anti-cancer drug for humans. Attempts to improve curcumin’s shortcomings such as a curcumin derivative, modification, analog, or pro-drug, have been extensively tested but without success. This is partly because curcumin has multifunctional activities^7^ and derivatives were created without knowledge of the precise effects of the anti-tumorigenic activity of curcumin. We previously found that curcumin binds to ROS metabolic enzymes, controls intracellular ROS levels, and specifically induces senescence and death in tumor cells^10^. In fact, some curcumin derivatives fail to maintain this activity. In this study, we characterized PGV-1, an analogue of curcumin^16^, and found that PGV-1 induced ROS with much earlier kinetics than curcumin and was superior to curcumin in anti-tumorigenic activity. Compared to curcumin, PGV-1 was effective at a lower concentration, had more rapid activity, arrested the cell cycle at a more specific point (prometaphase), and suppressed tumor formation more efficiently *in vivo* via p.o. Importantly, application of PGV-1 and curcumin resulted in no side effects. Furthermore, PGV-1 is superior to conventional anti-cancer drugs. In the case of CML, for example, 0.24 μM STI-571 inhibited proliferation of CML-derived K562 cells *in vitro*^19^, but K562 cells treated with 7.5 μM STI-571 for 2 days started to proliferate again after removing the drug^10^, whereas treatment with 0.8 μM PGV-1 continuously inhibited the growth and increased the population of dead cells. In an *in vivo* situation, PGV-1 at the dose of 20 mg/kg BW PO/2 days efficiently suppressed tumor formation in mice, which is equivalent to 90–120 mg PO/day in human^20^, while STI-571 at the dose of 400 mg PO/day is administrated in human to cure CML^21^.Taken together, because of the high drug efficacy and low side effects in animals, PGV-1 is an ideal anti-cancer drug candidate, and we propose that PGV-1 should be pharmaceutically developed as an orally administered drug for cancer.

We investigated the putative molecular activity of curcumin/PGV-1 on ROS metabolic enzymes: competition with co-factors for binding to the enzymes, thereby inhibiting the enzymatic activity of ROS scavengers. In the case of GST-P1 for example, PGV-1 competes with GSH for the binding to GST-P1 to inhibit its enzymatic activity. This result suggests that the ability of PGV-1 to inhibit GST-P1 activity in the cell depends on the amount of GSH available in the cell. Tumor cells often contain high levels of ROS^22^, which benefit tumor cells for their proliferation and high rate of mutagenesis^22^, resulting in a constitutive consumption of intracellular anti-oxidant such as GSH. Therefore, the actually available amount of GSH estimates much less than it appears. Thus, it is suggested that PGV-1 inhibits GST-P1 activity more effectively in tumor cells than in normal cells. This may be the reason why PGV-1 selectively suppresses tumor cell proliferation with few effects on normal cells.

It remains to be clarified whether the enzymes shown in this study (GST-P1, NQO1, NQO2, GLO1, and AKR1C1) are the only PGV-1-target proteins functioning in the ROS metabolic pathway. In the case of curcumin, we previously tried overexpression of wild-type enzymes (co-expression of 7 enzymes at the same time) and examined, but overexpressed cells were still sensitive to the inhibitory action of curcumin^10^. In the same analogy, we suspect that overexpression of wild-type enzymes in the cell will not confer full-resistance to PGV-1. In another attempt, designing the mutant enzymes that do not bind to PGV-1 will be a good approach, but is also a difficult task to perform because the PGV-1-binding site locates near the region required for the co-enzyme-binding. For example, Tyr7 and Asp98 in GST-P1 (Fig. 3c) are involved in the interaction with PGV-1, but are required for the enzymatic activity and interaction with GSH (UniProt database), respectively, suggesting that the substitution of these amino acids will negate the GST activity and the binding to GSH as well as the PGV-1 binding. Other strategies are needed to settle down this issue.

In *in vitro* assays, binding and inhibition of PGV-1 to ROS metabolic enzymes were comparable to that of curcumin, but, in cultured cells, PGV-1 exhibited much stronger growth inhibitory activity and increased ROS levels at much earlier time points than curcumin. This may be because PGV-1 is more efficiently incorporated into the cell, is more stable in the cell, or is more accessible to the target molecule in the cell than curcumin. Or, otherwise, PGV-1 may have its own (specific or selective) target molecules. Therefore, characterization of PGV-1-target enzymes will be an obvious next issue to be solved. The pulldown assay using PGV-1 beads and cancer cell lysates followed by the MASS analysis may reveal PGV-1-interacting proteins.

Curcumin suppresses cell cycle progression through G2/M phase^23–27^, but also negatively controls other phases of the cell cycle (G1 and S phases)^10^. In contrast, the activity of PGV-1 was restricted to G2/M phase. Among many anti-cancer drugs, vinca alkaloids (vincristine and vinorelbine)^28^ and taxanes (paclitaxel and docetaxel)^29,30^ inhibit progression through M phase. Because these anti-mitotic agents inhibit microtubule function^31–33^, which prevents axonal transport in neurons, neurotoxicity is a specific side effect in addition to the common side effect, myelosuppression^33^ (the LD50 of vincristine and paclitaxel are 3 mg/kg and 30 mg/kg, respectively). In contrast, PGV-1 had few side effects at the concentration (20 mg/kg) at which tumor formation was suppressed; no decrease in body weight, WBC, and RBC, and no signs of peripheral nerve disorder. These results suggest that the target molecule of PGV-1 (and curcumin) is different from tubulin, and PGV-1 arrests cells at prometaphase other than by inhibiting the polymerization/depolymerization of tubulin. Both PGV-1 and curcumin increase intracellular ROS levels, but the effects of PGV-1 were more rapid than curcumin, which may contribute to a specific cell cycle arrest in M phase. However, PGV-1 may also act through different targets, which are more specific/selective to PGV-1 than curcumin. Whatever the target, its function is specifically connected to tumorigenesis, and identification of the target of PGV-1 may help develop new anti-cancer therapies.

After 24 hours post PGV-1-addition, flow cytometric data (Fig. 2a,b) showed that >80% of cells arrested in G2/M phase, mitotic indices (Fig. 2c,d) showed that ca 40% of cells were in M phase, and the β-gal assay (Fig. 2g,h) showed that >50% of cells underwent senescence. These data indicate that PGV-1-treated cells entered senescence with 4 N DNA content. Recently, several reports demonstrated that cells enter senescence with 4 N DNA content^34–36^. Therefore, PGV-1 could be a useful tool to investigate how cells enter senescence from G2/M phase.

The molecular action of PGV-1 was likely responsible for its effectiveness on anti-tumorigenicity; PGV-1 first raised intracellular ROS levels at an early time point (ca 2 hr), arrested the cell cycle at prometaphase, induced senescence to prevent return to the cell cycle, and induced cell death. PGV-1 prevented the growth of various human cancer cells and leukemic cells. Cancer cells contain relatively high ROS levels^12,13^, which makes cancer cells a better target for PGV-1 and curcumin than normal cells. In addition, cancer cells with mutations in genes encoding tyrosine kinase receptors and Ras proteins may be good targets for PGV-1-mediated cancer therapy because constitutive activation of growth signaling pathways increases ROS levels^22^. In conclusion, PGV-1 was highly potent against cancer and may be used for highly malignant cancers, such as pancreatic cancer and lung cancer, because these cancer cells have frequent mutations in EGF receptors and K-Ras.

## Materials and Methods

### Compounds

Curcumin ((1E,6E)-1-(4-hydroxy-3-methoxyphenyl)-7-(3-methoxy-4-methylphenyl) hepta-1,6-diene-3,5-dione) (the purity is ≥93%), and PGV-1 ((2E,5E)-2-[(4-hydroxy-3,5-dimethylphenyl)methyldene]-5-[(3-methoxy-4,5-dimethylphenyl)methylidene] cyclopentan-1-one) (the purity is ≥99.8%), were obtained from the Curcumin Research Center (CRC), Faculty of Pharmacy, Universitas Gadjah Mada. Curcumin was also purchased from Sigma and Merck.

### Cell culture and transfection

293T human embryonic kidney cells, human cancer cell lines (U-87, MCF-7, HeLa, 293T, AN3CA, Mia Paca-2, PANC-1), and early-passage (p3) mouse embryonic fibroblasts (MEFs) were cultured in Dulbecco’s modified Eagle’s medium (DMEM) supplemented with fetal bovine serum (FBS) and antibiotics as described^10^. Transfection was carried out by the calcium phosphate-DNA precipitation method^37^. CML-derived K562 cells were maintained and the growth curve analysis was performed as described^10^. Where indicated, cells were washed in PBS, and re-plated in fresh medium without curcumin and PGV-1. Viable and dead cells were counted using the trypan blue exclusion method. In some cases, cells were fixed on slide glass by cytospin and inspected after staining with May-Grünwald Giemsa solution (Merck).

### Cell cycle analysis and β-gal assay

In the cell cycle analysis, cells were incubated in a solution containing 0.1% sodium citrate and 0.1% Triton X-100 containing 50 μg/mL of propidium iodide and treated with 1 μg/mL of RNase at room temperature for 30 min as described previously^10^. Fluorescence from the propidium iodide-DNA complex was measured with a FACSCalibur flow cytometer (Becton Dickinson).

For measuring the mitotic index, cells were treated with a hypotonic buffer (5.6% KCl) for 6 min at room temperature, fixed in a fixative solution (3:1 methanol:acetic acid), and dropped on a sterile microscope slide (mitotic spread). Chromosomal DNA was stained with Hoechst 33342, and observed by confocal microscopy. Mitotic and total cells were counted. In some cases, cells were collected by cytospin, stained with May-Grünwald Giemsa solution, and observed by phase-contrast microscopy.

In the senescence associated-β-galactosidase (SA-β-gal) assay, cells were fixed in 0.2% glutaraldehyde or 2% PFA, incubated for 16–24 hr in X-Gal solution containing 0.2% X-Gal, 2 mM MgCl_2_, 5 mM K_4_Fe(CN)_6_, and 5 mM K_3_Fe(CN)_6_, and viewed with a phase-contrast microscope as described previously^10^.

### Measurement of intracellular ROS levels

Cells were stained using the Cellular Reactive Oxygen Species Detection Assay Kit (Deep Red Fluorescence, Sigma) according to the manufacturer’s instructions or were stained with DCFDA (2′,7′-dichlorofluorescin diacetate), and analyzed with a FACSCalibur flow cytometer (Becton Dickinson) as described previously^10^.

### Immobilization of curcumin and PGV-1 on epoxy-sepharose beads and a pull-down assay

Curcumin and PGV-1 were immobilized on epoxy-activated sepharose beads as described previously^10^, and the pulldown assay was performed with the cell lysate containing the HA-tagged protein followed by immunoblotting using a mouse monoclonal antibody to hemagglutinin (HA) peptide epitopes (12CA5) as described^10,38^.

### Plasmid construction

Amplification of cDNAs by PCR and generation of the expression vectors for CBR1, PRDX1, NQO1, NQO2, GLO1, AKR1C1, and GST-P1 were described previously^10^. For cloning of GST-O1 cDNA, we used PCR primers 5′-TCTAGAACGATGTCCGGGGAGTC-3′ (forward) and 5′-GGATCCCTTTATTGCTGACTCCTGC-3′ (reverse).

### GST enzyme assay

6xHis-tagged GST-P1 recombinant protein was expressed in bacteria, affinity purified using Ni-NTA agarose beads (QIAGEN), eluted with 0.5 M imidazole, and dialyzed against PBS at 4 °C. Two micrograms of recombinant GST-P1 protein was incubated with curcumin and PGV-1 for 1 hr on ice, then mixed with reaction solution (0.1 M potassium phosphate buffer, pH 6.5) containing 2 mM glutathione reduced form (GSH) (Nacalai tesque) and 1 mM 1-chloro-2,4-dini-trobenzene (CDNB) (Sigma)^18^. To detect GSH-conjugated CDNB, absorbance of 340 nm was recorded every 10 sec for 5 min at 25 °C with a UV-1800 spectrophotometer (SHIMADZU). The Km and Vmax values were determined from direct plots of velocity versus substrate concentration, and are presented along with their standard errors.

### Animal experiments

Mice were kept in specific pathogen-free conditions (the Animal Experimentation Facility of NAIST), and all methods were performed in accordance with NAIST guidelines and regulations. The experimental protocols used in this study were approved by the NAIST institutional and licensing committees.

Subcutaneous (s.c.) injection of K562 cells, intra-peritoneal (i.p) administration of the compound, peripheral blood tests, and measurement of tumor weights were described previously^10^. Oral administration (per os, p.o.) was also used with the same concentration of the compound as used in i.p administration in this study.

### Statistical analysis

Data are shown as the mean ± S.D., SPSS 24 was used to conduct statistical analyses, and the Student’s *t*-test was used to examine the significance of differences between two experimental conditions as described previously^10^. The values for the significance of differences (p values, *p < 0.05, **p < 0.01, ***p < 0.001, and ****p < 0.0001) were added to every figure.

### Molecular docking analysis

To predict the interacting structures of the ligand PGV-1 or curcumin with target proteins NQO1, NQO2, GLO1, AKR1C1, and GST-P1, we searched Protein Data Bank (PDB)^39^ for high-resolution structures complexed with their specific co-factors. We selected the NQO1 structure at 1.7 Å resolution (PDB ID: 1D4A^40^, chains A and B), the NQO2 structure at 1.2 Å resolution (PDB ID: 4FGL^41^, chains A and B), the GLO1 structure at 1.72 Å resolution (PDB ID: 1QIP^42^, chains A and B), the AKR1C1 structure at 1.59 Å resolution (PDB ID: 1mrq^43^, chain A), and the GST-P1 structure at 1.19 Å resolution (PDB ID: 5j41^44^, chains A and B) as target structures. For each enzyme, a docking study was performed as follows: Because PGV-1 binding sites on the enzymes were unknown, we first predicted them using two criteria. First, a pocket (concave area on a molecular surface) search on the enzyme structure was conducted using fpocket2 software^45^ with default parameters. The top five pockets ranked by fpocket2 score were considered candidate sites for PGV-1 binding. Second, a co-factor binding site, which was composed of amino acid residues containing atoms located within 4.0 Å of the co-factor molecule, was considered as another candidate site of PGV-1 binding. In total, six candidate PGV-1 binding sites were obtained for each enzyme. After all co-factors, other ligands, and water molecules were removed from structure coordinates, the enzyme structure was preprocessed using the Protein Preparation wizard in the Schrödinger Release 2018–2 (Schrödinger, LLC, New York, NY, 2018). The preprocess was composed of assigning bond order, adding hydrogen atoms, optimizing H-bond networks, and restraining energy minimization with OPLS3 force field^46^. Using the preprocessed structure, receptor energy grids were generated within a 18 × 18 × 18 Å box centered on each candidate PGV-1 binding site. Furthermore, we prepared another receptor energy grid generated within a 18 × 18 × 18 Å box centered on the co-factor molecule using the preprocessed structure complexed with the co-factor molecule. The PGV-1 structure, which was the most stable conformation generated by Balloon software^47^ with options–nconfs 20,–nGenerations 300, and–randomSeed 201807, was docked using Glide in the standard precision (SP) mode^48^. Up to 10 poses were obtained for each receptor energy grid. Using all poses obtained from the SP mode as initial structures of PGV-1, pose refinement with the extra precision (XP) mode^49^ was performed. The docking pose with the highest XP score was selected as the final docking pose.

The docking of curcumin was also performed using the same procedure as PGV-1 docking except the energy grid size was 20 × 20 × 20 Å.

## Supplementary information

Supplementary Information
