# Supplementary material for: Pentagamavunon-1 (PGV-1) inhibits ROS metabolic enzymes and suppresses tumor cell growth by inducing M phase (prometaphase) arrest and cell senescence
Source: Sci Rep. 2019 Oct 16;9:14867. doi: 10.1038/s41598-019-51244-3 (PMC6795878; doi:10.1038/s41598-019-51244-3)

Pentagamavunon-1 (PGV-1) inhibits ROS metabolic enzymes and suppresses tumor cell growth by inducing M phase (prometaphase) arrest and cell senescence.

Beni Lestari[1], Ikuko Nakamae[1], Noriko Yoneda-Kato[1], Tsumoru Morimoto[2], Shigehiko Kanaya[3], Takashi Yokoyama[1], Masafumi Shionyu[4], Tsuyoshi Shirai[4], Edy Meiyanto[5], and Jun-ya Kato[1*]

[1]Laboratory of Tumor Cell Biology, Division of Biological Science, [2]Laboratory of Synthetic Organic Chemistry, Division of Materials Science, [3]Laboratory of Computational Systems Biology, Division of Information Science, Graduate School of Science and Technology, Nara Institute of Science and Technology, Nara, Japan, [4]Nagahama Institute of Bio-Science and Technology, Nagahama, Japan, [5]Cancer Chemoprevention Research Center, Faculty of Pharmacy, Universitas Gadjah Mada, Yogyakarta, Indonesia.

Figure S1 - Related to Figure 2

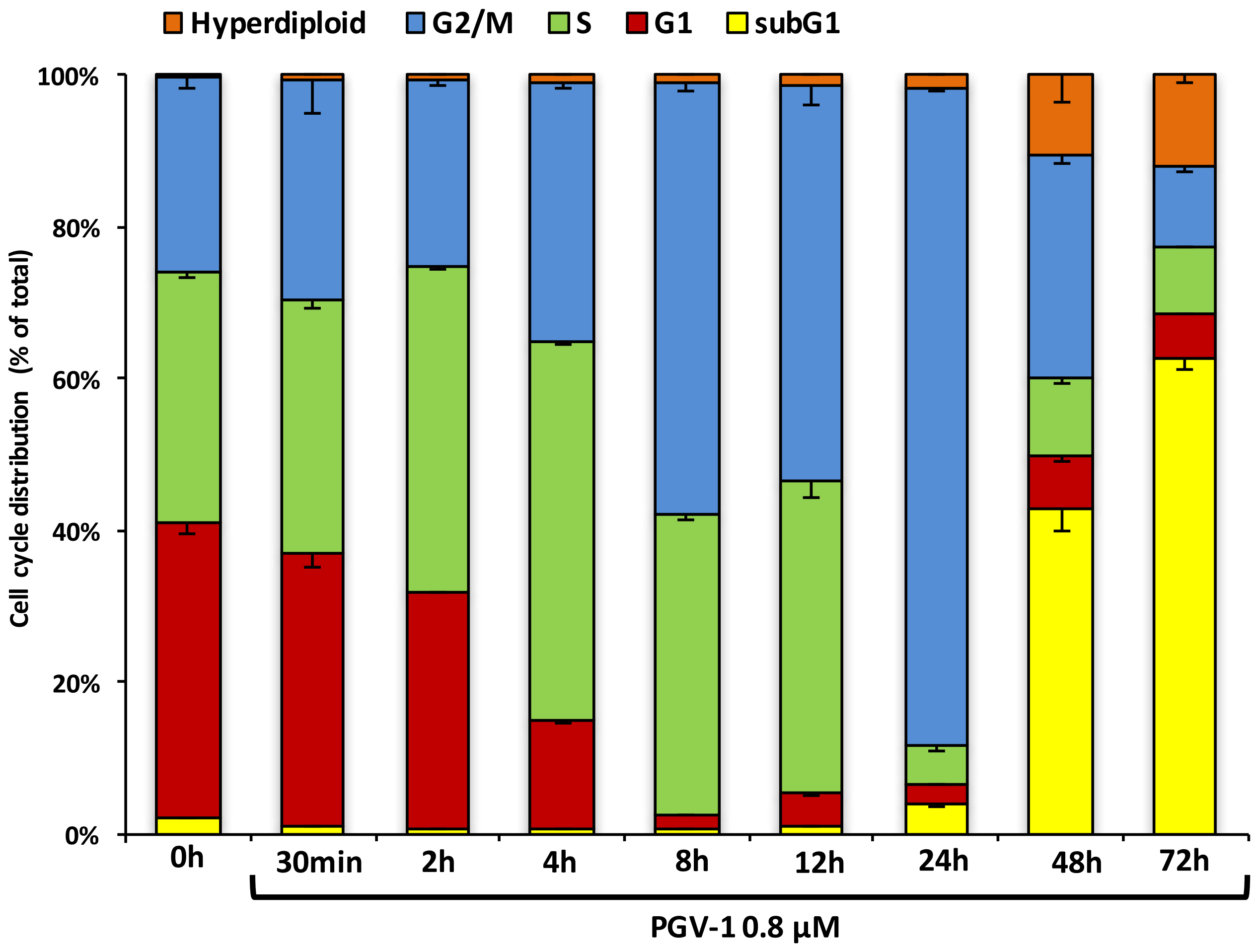

Legend for Supplementry Fig. S1

K562 cells (5 x 10^5 cells/mL) were treated with PGV-1 (0.8 μM) for 0.5, 2, 4, 8, 12, 24, 48, and 72 hr, and then subjected to cell cycle analysis. The percentages of cells in each phase are shown as the mean ± SD from three independent experiments.

Figure S2 – Related to Figure 3

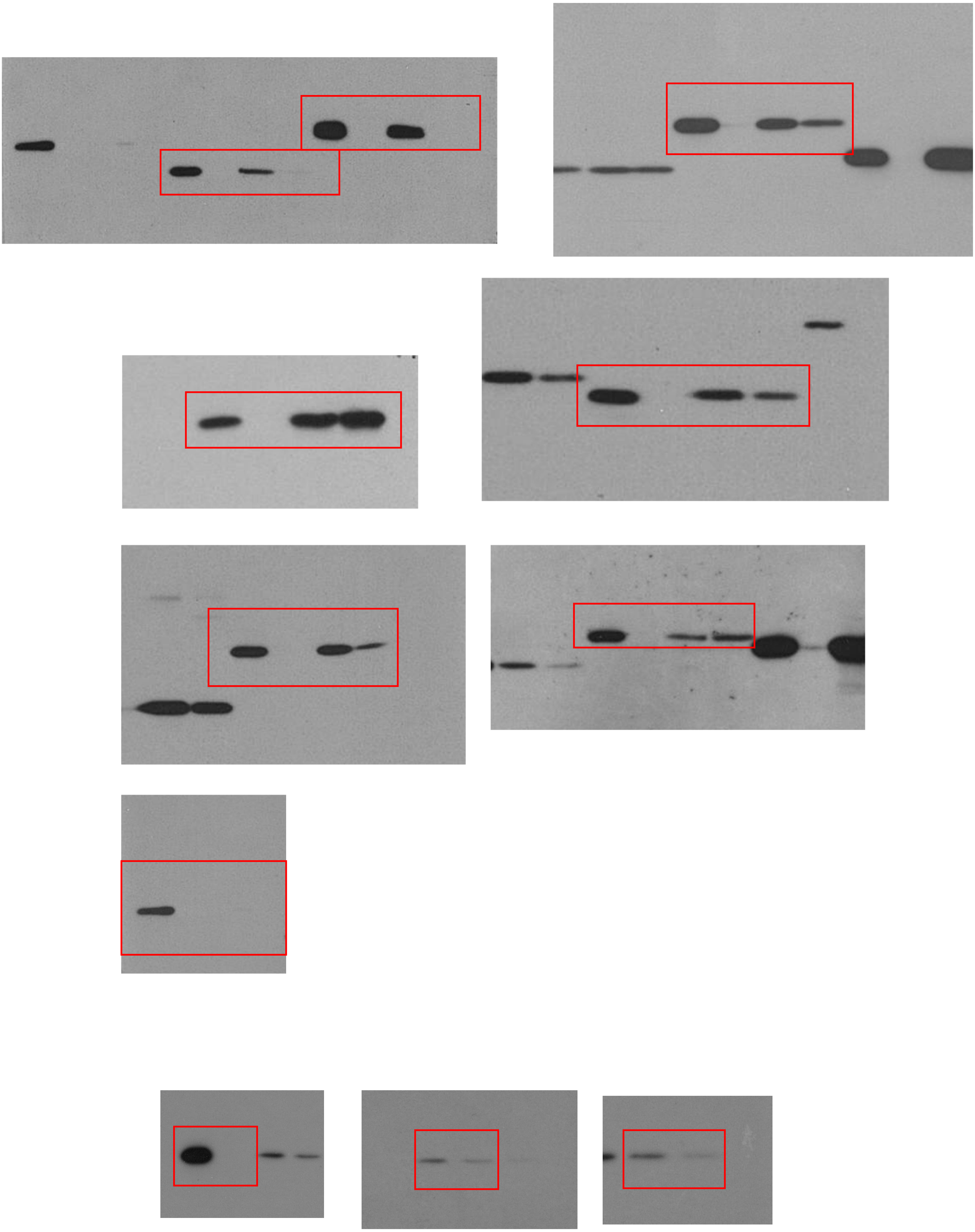

Supplement: Supplementary file 1 — Supplementary Information [file 41598_2019_51244_MOESM1_ESM.pdf]
